# Changes in Effective Connectivity by Propofol Sedation

**DOI:** 10.1371/journal.pone.0071370

**Published:** 2013-08-19

**Authors:** Francisco Gómez, Christophe Phillips, Andrea Soddu, Melanie Boly, Pierre Boveroux, Audrey Vanhaudenhuyse, Marie-Aurélie Bruno, Olivia Gosseries, Vincent Bonhomme, Steven Laureys, Quentin Noirhomme

**Affiliations:** 1 Coma Science Group, Cyclotron Research Centre and Neurology Department, University and University Hospital of Liège, Liège, Belgium; 2 Cyclotron Research Centre, University of Liège, Liège, Belgium; 3 Department of Electrical Engineering and Computer Science, University of Liège, Liège, Belgium; 4 Department of Anesthesiology and Reanimation, University Hospital of Liège, Liège, Belgium; Hangzhou Normal University, China

## Abstract

Mechanisms of propofol-induced loss of consciousness remain poorly understood. Recent fMRI studies have shown decreases in functional connectivity during unconsciousness induced by this anesthetic agent. Functional connectivity does not provide information of directional changes in the dynamics observed during unconsciousness. The aim of the present study was to investigate, in healthy humans during an auditory task, the changes in effective connectivity resulting from propofol induced loss of consciousness. We used Dynamic Causal Modeling for fMRI (fMRI-DCM) to assess how causal connectivity is influenced by the anesthetic agent in the auditory system. Our results suggest that the dynamic observed in the auditory system during unconsciousness induced by propofol, can result in a mixture of two effects: a local inhibitory connectivity increase and a decrease in the effective connectivity in sensory cortices.

## Introduction

Propofol provides a reversible pharmacological manipulation of consciousness and is widely used as a hypnotic anesthetic agent [1]. Nevertheless, the precise mechanism that induces unconsciousness during propofol sedation is still unclear. Theoretically, hypnotic agents may have two main ways of inducing an alteration of consciousness: by suppressing the activity in relevant regions involved in the consciousness processes or by altering the brain communication mechanisms [1]. The first process has been extensively studied by computing regional differences in neuronal activity between wake and unconsciousness states using electrophysiological or functional neuroimaging measurements [2]–[4]. The second mechanism, i.e., the potential brain communication breakdown, is still poorly understood [5].

Several studies have shown propofol-induced decreases in neuronal activity in fronto-parietal associative networks including precuneus and thalamus [6]–[11]. These findings not only suggest a strong relationship between suppression of neuronal activity and loss of consciousness [1], but also a possible alteration of the communication mechanisms in thalamo-cortical networks [12]. However, measurments of neuronal deactivation only indirectly point to a potential communication breakdown [13]. Recently, functional connectivity has been used to measure the covariance observed in the activity of different brain regions under propofol using functional magnetic resonance imaging (fMRI) resting state. Boveroux et al. [14] found that propofol induces a sensible reduction of the functional connectivity within higher-order fronto-parietal cortices during deep sedation, while connectivity in low-level sensory networks was relatively preserved. A more quantitative resting state connectivity assessment was performed by Schrouff et al. [15], who showed that the loss of consciousness caused by propofol was associated with an alteration in the capacity of the brain to integrate information as well as with a segregation of the fronto-parietal brain networks during unconsciousness. In a related study, Mhuircheartaight et al. [10] reported that the failure to respond to auditory stimuli during propofol sedation was associated to a decrease in functional connectivity between putamen and other brain regions. Additionally, these authors reported a relative preservation of the thalamo-cortical functional connectivity. All these evidences suggest an important role of the cortico-cortical communication mechanisms for the generation of propofol induced loss of consciousness. Nevertheless, these studies are only based on observing changes in statistical relationships among indirect observations of neuronal activity. Moreover, the employed functional connectivity approaches do not provide any mechanistic explanation about the physiological phenomena involved in the alteration of consciousness [16].

Effective connectivity is a different alternative to study the brain communication mechanisms. The goal of this type of connectivity analysis is to characterize the causal influence that one neuronal system exerts over another [16]. The main difference between functional and effective connectivity is that the latter quantifies the directional influences at the neuronal level, based on an underlying causal model of neuronal activity, i.e., a model driven approach. In contrast, functional connectivity corresponds to a data driven approach. One of the most extensively used frameworks to study effective connectivity is Dynamical Causal Modeling (DCM) [17]. This framework considers parametric causal models of the neuronal dynamics, which are used to simulate indirect neuronal measurements usually captured by neuroimaging techniques, such as fMRI and electroencephalography (EEG). DCM brain models include connectivity parameters, specifying the existence and strength of directional connection and a set of biophysical parameters for mapping neuronal activity to experimental measurements [13], [17]. By differentially specifying the model connectivity parameters, it is possible to define different models (hypotheses) concerning the generation of neuronal activity [18]. DCM thus uses the experimental measurements of the neuronal activity (for example, the BOLD signal) to infer the most plausible hidden biophysical quantities (for instance, hemodynamic parameters in the case of BOLD), and other more abstract parameters usually related with connectivity strengths. DCM also provides a complexity/fitting estimation for the fitted model based on a bound, the free energy, of the model log-evidence [19]. Model evidence can be used in a Bayesian Model Selection (BMS) procedure to compare different hypotheses and selecting the most plausible among those considered and given the experimental observations [20]. In addition, different models can be summarized in a single parametric model using Bayesian Model Average (BMA), resulting in a model independent measure of the underlying brain dynamics [21]. In summary, DCM is a framework to compare several hypotheses about causal neuronal connectivity using experimental observations of the neuronal processes.

The aim of the present study was to investigate the changes in effective connectivity correlating with the alteration of consciousness across several levels of propofol-induced sedation in healthy humans. We used DCM to study the dynamics of a neuronal system involved during the processing of complex auditory stimuli. We studied four different consciousness states: wakefulness, mild sedation, unconsciousness and recovery. Our main hypothesis was that the effect of propofol-induced alteration of consciousness in this cortical system would result from a mixture of two actions: a local self-inhibitory connectivity increase and a decrease in effective connectivity within auditory sensory cortices. In order to build a realistic model space for our analysis, we constructed several connectivity models on top of different fMRI-DCM generative models. These models included a deterministic model with causal interactions [17], a stochastic model that contemplates physiological noise [22] and one including excitatory (glutamatergic) and inhibitory (GABAergic) local effects [23]. The models were related to the interaction among regions located in the auditory system. Previous studies have shown that during unconsciousness and altered consciousness states the auditory system can exhibit changes in connectivity among these regions [24]–[28]. This is a key feature of these mental states and can in general affect brain activity [29], [30]. In this sense, this system will provide a good model to investigate a common mechanism involved in unconsciousness and relevant for the whole brain. In this study, we further investigated the basis of these phenomena by focusing in the effective connectivity. Using this strategy, we expect to provide general explanations of the unconsciousness phenomena that can be translated to other less controlled loss-of-consciousness scenarios, such as, disorders-of-consciousness or epileptic seizures [1], [31] where these phenomena is also present.

## Materials and Methods

### Participants

The study was approved by the Ethics Committee of the Medical School of the University of Liège (University Hospital, Liège, Belgium). The subjects provided written informed consent to participate in the study. Eleven healthy right-handed volunteers participated in the experimentation (10 women and 1 man; age range, 20–31 yr; mean age ±SD, 24±3.5 yr). None of the subjects had a history of head trauma or surgery, mental illness, drug addiction, asthma, motion sickness, or previous problems during anesthesia.

### Sedation protocol

We used the protocol previously described by Boveroux et al. [14]. Subjects fasted for at least 6 hours from solids and 2 hours from liquids before anesthesia. During the study and the recovery period, electrocardiogram, blood pressure, pulse oximetry (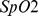), and breathing frequency were continuously monitored (Magnitude 3150M; Invivo Research, Inc., Orlando, FL). Propofol was infused through an intravenous catheter placed into a vein of the right hand or forearm. An arterial catheter was placed into the left radial artery. Throughout the study, the subjects breathed spontaneously, and additional oxygen (5 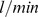) was given through a loosely fitting plastic facemask. Sedation was achieved using a computer-controlled intravenous infusion of propofol (Alaris® TIVA; Carefusion, San Diego, CA) to obtain constant effect-site concentrations. The propofol plasma and effect-site concentrations were estimated using the three-compartment pharmacokinetic model of Marsh et al. [32]. After reaching the appropriate effect-site concentration, a 5-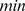 equilibration period was allowed to insure equilibration of propofol repartition between compartments. Arterial blood samples were then taken immediately before and after the scan in each clinical state for subsequent determination of the concentration of propofol and for blood-gas analysis. The level of consciousness was evaluated clinically throughout the study with the scale used by Ramsay et al. [33]. The subject was asked to squeeze the hand of the investigator. She/he was considered fully awake or to have recovered consciousness if the response to verbal command ( “squeeze my hand!”) was clear and strong (Ramsay 2), in mild-sedation if the response to verbal command was clear but slow (Ramsay 3), and in deep sedation/unconsciousness if there was no response to verbal command (Ramsay 5–6). For each consciousness level assessment, Ramsay scale verbal commands were repeated twice.

### Task and functional data acquisition

The first names of the participants as well as familiar names (mother or father's name) were used as stimuli. Names were recorded by female and male voices, repeated with three different intonations in order to avoid habituation effect, and were presented in blocks of 25 seconds with the same name repeated every 2 seconds within the block. A 20 seconds silent period followed each block. There were 8 blocks of each name for a total of 16 blocks arranged in a randomized order. Each participant heard his own name 80 times and heard each of the familiar names 80 times per condition in passive listening task. fMRI acquisition was performed in four conditions, i.e., in four clinical states: normal wakefulness (Ramsay 2), mild-sedation (Ramsay 3), unconsciousness (Ramsay 5), and recovery of consciousness (Ramsay 2). The temporal order of mild-sedation and unconsciousness conditions was randomized. Each scan session lasted approximately 14 min.

Functional images were acquired on a 3 Tesla Siemens Allegra scanner (Siemens AG, Munich, Germany; Echo Planar Imaging sequence using 32 slices; repetition time  = 2460 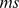, echo time  = 40 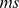, field of view  = 220 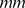, voxel size  = 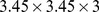 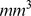, and matrix size  = 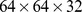 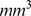). A high resolution T1 image was also acquired in each volunteer at the end of the whole experiment to provide a an image of the subjects cerebral anatomy. During data acquisition, subjects wore headphones. The most comfortable supine position attainable was sought to avoid painful stimulation related to position.

### Dynamic causal modeling

The proposed DCM analysis scheme proceeded with the following steps: (1) definition of an anatomical network of contributory regions, (2) definition of a set of models based on variations of the connectivity architecture within this network, (3) extraction of the BOLD fMRI time series from the network regions for each subject, (4) estimation of the models parameters for the different fMRI-DCM generative models using the observed data, (5) comparison of the different families of fMRI-DCM generative neuronal dynamics, (6) comparison of individual models using the free energy estimate of the model log-evidence in each consciousness state, and finally, (7) comparison of connectivity parameters.

#### Dynamic causal modeling

The general goal of DCM is to provide mechanistic explanations, in terms of effective connectivity, for the local effects observed in a conventional univariate analysis [16]. For this reason, DCM runs on a set of contributory regions on which the estimation is performed [17]. In the present study we modeled both own name and names as one single auditory entry.

A General Linear Model (GLM) analysis was performed to select regions involved in the auditory name processing. SPM8 (http://www.fil.ion.ucl.ac.uk/spm, Version 4010 (SPM8), 21-Jul-10). (Welcome Trust Centre for Neuroimaging, University College London, UK) was used for analysis, preprocessing and DCM computations. Images were realigned, normalized to a standard EPI template, and smoothed with a 3D-Gaussian kernel of 8 mm [34], [35]. The first three fMRI volumes were removed for signal equilibration. A normalization of the EPI mean image to the EPI template provided the parameters for normalizing the EPI scans. Regressors of the design matrix were created by convolving boxcar stimulus functions with a canonical hemodynamic response function. Head movements formed six additional regressors, three describing the rotation and three describing the translation of the subjects head during the functional scan. Linear contrasts of estimated parameters were created for each participant. A t-contrast testing for the main effect of names was built for each subject in each fMRI session. Finally, a random-effect group analysis was performed in wakefulness to detect activation to names. For the present study, the group SPM results showed activation in the primary and associative auditory cortices in the right dorsolateral prefrontal cortex (see section 0). We identified and selected three areas over which our hypotheses were constructed: Heschl's Gyrus [HG-(44,−25,14) 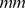], Superior Temporal Gyrus [STG-(63, −12, −3) 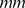] and Middle Frontal Gyrus [MFG-(38,2,48) 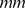]. HG and STG have been extensively identified as part of auditory cortex even activating during propofol sedation [8]. MFG has also been consistently identified in several name processing experiments in different conditions, including fluctuating states of consciousness [28], [36], [37]. In addition, MFG activation has been previously observed during attention-related tasks [38], [39]. A similar architecture involving middle temporal gyrus, inferior frontal gyrus and MFG was also previously proposed for a DCM analysis in idiomatic sentence processing [40]. Left HG and STG were also found in our analysis, however there was no evidence of activation in left MFG. For this reason and to keep our model as simple as possible, the left auditory subsystem was not modeled here. The specific areas for each participant were identified based on the coordinates of the peak activation obtained in the group analysis centering individually on the local activation maximum closest to each peak of interest. The selected local maximum was constrained to lie within 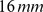 (twice the width of the Gaussian smoothing kernel) of the group peak coordinates and within the same anatomical gyrus/sulcus as the group activation. Data were extracted for each session separately within each region and adjusted to the F-contrast (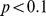 uncorrected) of each subject. Significant activation was observed for all the subjects in the selected regions. Across the 11 subjects, the mean (SD) of the MNI coordinates of the selected regions were: HG: 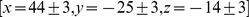, STG: 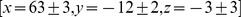 and MFG: 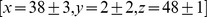. The specific areas for each participant were identified based on the coordinates of the peak activation obtained in the group analysis centering individually on the local activation maximum closest to each peak of interest. The selected local maximum was constrained to lie within 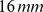 (twice the width of the Gaussian smoothing kernel) of the group peak coordinates and within the same anatomical gyrus/sulcus as the group activation. Data were extracted for each session separately within each region and adjusted to the F-contrast of each subject. Eigenvectors (i.e., time series) were extracted from each subject using the individual activation maps thresholded at p <0.05, uncorrected. Significant activation was observed for all the subjects in the selected regions. Across the 11 subjects, the mean (SD) of the MNI coordinates of the selected regions were: HG: 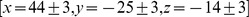, STG: 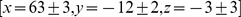 and MFG: 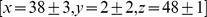.

#### Network specification

**Figure 1:**
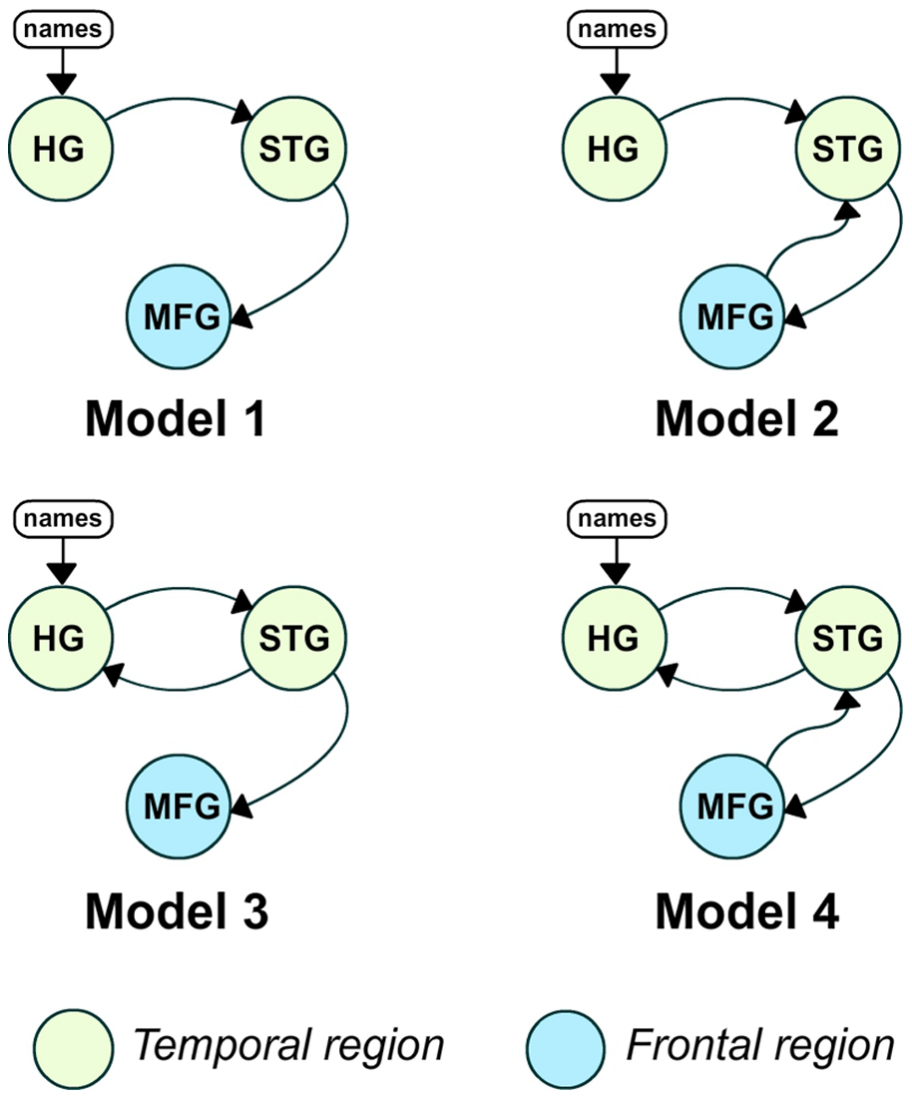
DCM models proposed for the processing of an auditory stimulus.

Our DCMs encompased the right Heschl's Gyrus (HG), superior temporal gyrus (STG) and middle frontal gyrus (MFG). The input area was located in the primary auditory cortex (HG) and was connected to areas that drive higher associative/attention areas (STG and MFG). Similar models have been proposed for DCM auditory studies [41], [42]. The proposed models consider one input: the name stimuli. The input region was HG, which can be physiologically considered as the natural entry for an auditory stimulation [41]. We considered 4 models (M1, M2, M3 and M4) with different forward and backward connections. Figure 1 shows the different proposed models.

Those models were based on the physiologically plausible auditory cascade configuration [41].

#### fMRI-DCM families

In practice, the DCM is implemented over a specific set of differential equations and a particular MIMO estimation algorithm [16]. Selection of the proper DCM implementation is a critical modeling issue closely related to the question of interest [43]. The most widely used DCM implementation in fMRI is the deterministic model with one-state per region [17]. In this implementation, direct causal influences among states are explicitly coded in the model and each contributory region is modeled by a single state variable associated to the inhibitory connection. In this work, some additional considerations have been taken into account to model the propofol effect.

Firstly, recent evidence shows that propofol enhances the activity of inhibitory 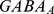 synapses locally [44], i.e., propofol increases the local inhibitory neuronal effect. This is an important issue because this inhibition could be improperly interpreted as a global decreasing in connectivity [45]. In addition, propofol could also change regions not explicitly modeled, for instance, thalamus or putamen [6], [10]. Unfortunately, the deterministic DCM framework cannot rule out the effect of these missing regions [43], [46]. To overcome these limitations we used two state-of-the-art fMRI-DCM extensions: the two-state DCM and the stochastic DCM.

The two-state DCM explicitly aims to capture local neuronal activity effect by using two local compartments: one for excitatory and another one for inhibitory subpopulations [23]. The use of this model will introduce additional complexity in the DCM, however, the explicit separation between inhibitory and excitatory mechanisms can help to isolate the potentiation of the 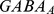 receptor, a characteristic effect expected for the propofol action [44]. This mechanism will be captured by the inhibitory subpopulation or in the mechanisms of interaction between inhibitory and excitatory subpopulations.

**Figure 2:**
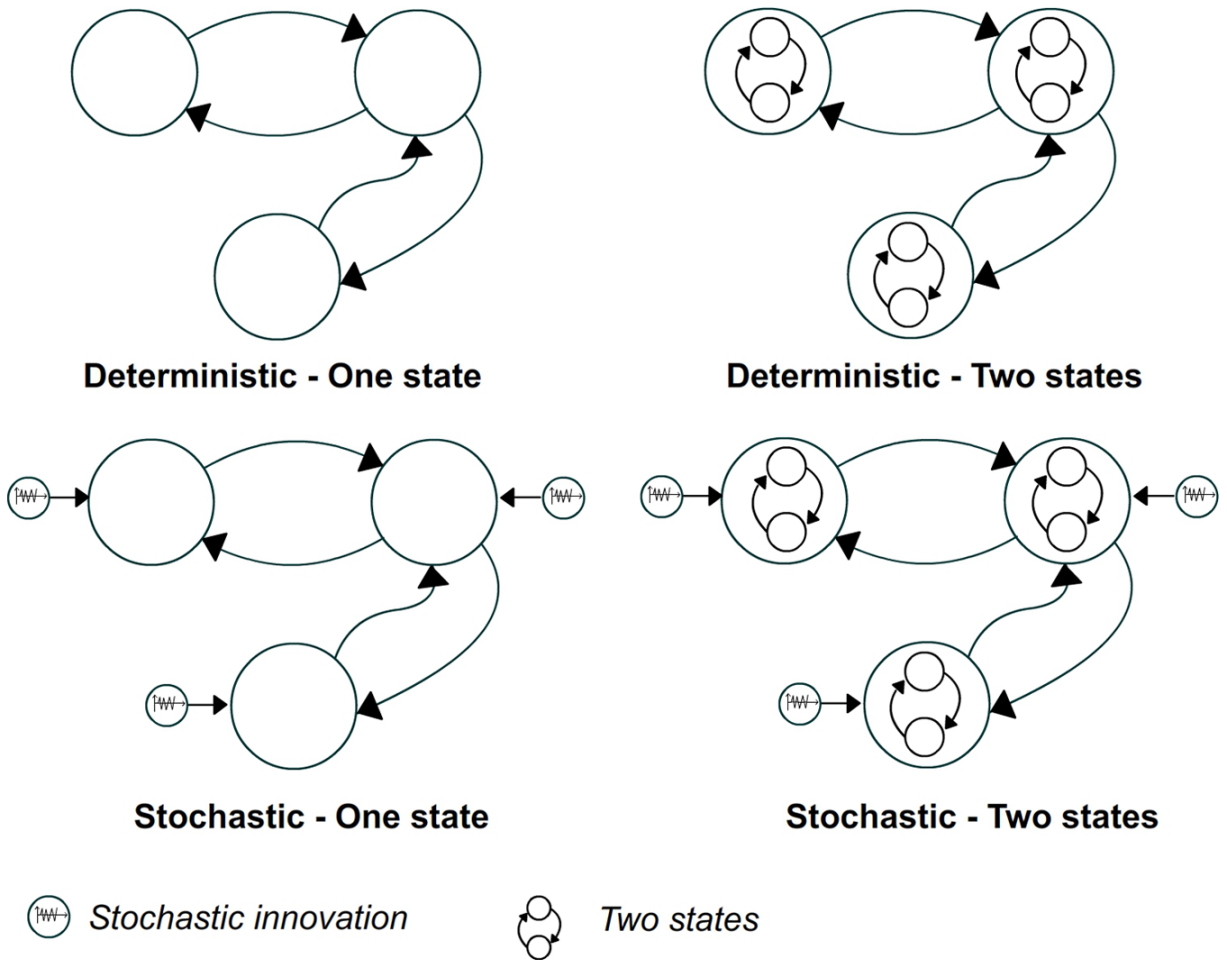
Different fMRI-DCM implementations proposed to capture the effect of the propofol agent. The deterministic one-state (DT1) provides the most simple neuronal model and the stochastic two-states (ST2) the most complex one.

The stochastic DCM was recently proposed as a DCM extension in which all indirect effects are modeled as a stochastic phenomenon. This model includes an “autonomous” hidden innovation term, which is added to the neuronal activity equation. This new term could explain uncontrolled exogenous influences to the system [22]. In this study we explored the effectiveness of these models to capture the propofol effect, by constructing and comparing four different DCM families: deterministic & one-state (DT1), deterministic & two-state (DT2), stochastic & one-state (ST1) and stochastic & two-state (ST2). Figure 2 illustrates the four DCM families explored.

The deterministic models were obtained from the stochastic ones by changing the driven noise log-precision, a parameter that controls the level of random fluctuations introduced to the states by the hidden innovation term [47]. We set this value of state-noise to 32, meaning a variation in the random fluctuations of 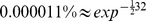 of the fMRI baseline values. This selection was previously used to generate deterministic data from a stochastic model [47]. All models were estimated using the dynamical expectation maximization (DEM) algorithm [48]. The 4 models were fitted with the data from each of the 11 controls in each of the 4 consciousness states using the 4 different DCM families. This provided the model log-evidence and posterior parameter estimates for each of the 704 (4×11×4×4) model fits.

### Bayesian model selection and averaging

Bayesian model selection (BMS) procedure was used to explore the set of models [19]–[21]. The aims of our BMS analysis were two fold: first, identify the most plausible DCM family to model the propofol action, and then, determine possible differences at the model level, i.e. architecture. For each consciousness state, we performed a family-wise BMS random effect analysis (RFX) comparing the four different DCM families: DT1, DT2, ST1 and ST2. RFX was used under the assumption that each subject could use a different model architecture [20]. Thereafter, we performed a RFX analysis at the model level on the winner family. First, we tested the hypothesis that a unique model was used in the four states of consciousness, for that we performed RFX over the whole set of models to pick the best architecture across the sedation levels [49]. In addition, we also tested the possibility of having different models in the different states of consciousess [43]. Additionally, we made inferences at the parameter level to provide a quantitative interpretation of the connectivity parameters and the possible changes induced by the anesthetic agent [43]. For this, we used BMA to compute a single posterior density over subjects per state of consciousness [21]. In BMA, the contribution of each model to the mean effect of each connectivity parameter is weighted by its evidence resulting in one parametric model that summarizes all averaged models. We averaged the four models (M1, M2, M3 and M4) of the winner DCM family for the complete population. Using this approach, we obtained a full sample-based (10000 samples) representation of the posterior density for each connectivity parameter [21]. Next, the significance of each connectivity parameter was examined. For that, we used the distribution of the discrete posterior density estimated in the BMA procedure to compute the cumulated probability of having connections lower than a certain threshold [17]. We considered significant connections the ones with BMA posterior probabilities 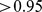 [50]. We also performed a one-tailed t-test on the ANOVA contrast for the self-inhibitory connections (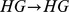, 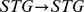 and 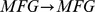), searching for a decrease-recovery relationship (contrast 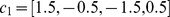) between the connectivity strength and the level of consciousness of the subjects across the four conditions (i.e., normal wakefulness, mild-sedation, unconsciousness, and recovery of consciousness) [14]. This contrast aims at capturing a decrease in connectivity strength during unconsciousness followed by a return to the initial connectivity during the recovery condition. A similar contrast (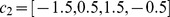) was applied on the connectivity parameters (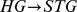, 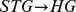, 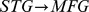 and 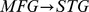) to search for increase-recovery effects, i.e., self-connectivity strength increases during unconsciousness followed by a restauration of the initial strength in the recovery of consciousness state. Finally, a contrast (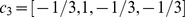) was used to examine the possibility of increased connectivity during mild-sedation, as recently observed for propofol and midazolam [51], [52]. Results were corrected for multiple comparisons and considered significant at 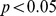.

## Results

### GLM analysis

Voxel-wise second-level analyses were performed for each consciousness state. Random effects analyses were used to detect activation to names. Table 1 reports the activated regions during the wake state (F-contrast, False Discovery Rate FDR, 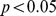).

**Table 1:** Brain regions showing main effects in wake state.

*Brain areas*	*R/L*	*BA*	*x (mm)*	*y (mm)*	*z (mm)*	*F-value* ^a^
*Sup. temporal gyrus^b^*	R	21	63	−12	−3	13.52
*Trans. temporal gyrus (Helsch's gyrus)^b^*	R	41	44	−25	14	11.28
*Sup. temporal gyrus*	R	22	55	−42	7	7.82
*Trans. temporal gyrus*	R	41	−40	−25	10	12.25
*Trans. temporal gyrus (Helsch Gyrus)*	L	42	−61	−15	10	10.89
*Insula*	L	13	−46	−36	18	7.75
*Sup. temporal gyrus*	L	22	−65	−48	15	7.12
*Sup. temporal gyrus*	L	22	−65	−46	6	4.45
*Prec. gyrus*	L	6	−30	0	39	6.07
*Mid. frontal gyrus^b^*	R	6	38	2	48	5.93
*Prec. gyrus*	R	6	51	0	50	5.48
*Mid. frontal gyrus*	R	8	51	6	44	5.41
*Inf. frontal gyrus*	R	45	53	18	5	5.05

Sup., superior; Trans., transverse; Mid., middle; Prec. precentral; Inf. inferior; L, left; R, right; BA, Brodmanns area. ^a^ FDR, 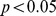 (random-effect analysis). ^b^ regions selected for DCM.

In mild sedation and deep sedation there was no evidence of significant activations to the stimuli. Table 2 reports significant regions activated during recovery (F-contrast, uncorrected, 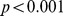).

**Table 2:** Brain regions showing main effects in recovery state.

*Brain areas*	*R/L*	*BA*	*x (mm)*	*y (mm)*	*z (mm)*	*F-value^a^*
*Sup. temporal gyrus*	R	22	50	−28	6	7.68
*Mid. temporal gyrus*	R	22	66	−32	4	6.75
*Sup. temporal gyrus*	R	9	64	−4	4	6.50
*Sup. temporal gyrus*	L	22	−44	−28	8	6.90
*Sup. temporal gyrus*	L	22	−62	−38	8	6.82
*Insula*	L	9	−54	−40	14	5.91
*Inf. frontal gyrus*	R	22	30	12	−18	6.39
*Mid. temporal gyrus*	L	22	−56	2	−8	6.02
*Mid. frontal gyrus*	R	9	−12	−38	24	5.46
*Sup. temporal gyrus*	R	22	66	−40	18	5.27
*Sup. temporal gyrus*	R	22	52	8	4	4.58
*Mid. frontal gyrus*	L	9	−28	16	28	4.35

Sup., superior; Mid., middle; Inf. inferior; L, left; R, right; BA, Brodmanns area. ^a^ uncorrected, 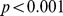 (random-effect analysis).

For the DCM construction three areas activated during the wake state were selected: Heschl's gyrus, superior temporal gyrus and middle frontal gyrus. Significant differences between the four conditions were explored by using a factorial design, one factor by state of consciousness. There was no evidence of differences between the four conditions neither at the whole brain nor at the regions of interest selected for the DCM construction. As observed in table 3.

**Table 3:** Brain regions showing significant changes when comparing wake and deep sedation.

*Brain areas*	*R/L*	*BA*	*x (mm)*	*y (mm)*	*z (mm)*	*F-value^a^*
*Pos. cingulate*	L	30	−22	−56	14	21.85
*Pos. cingulate*	L	30	−12	−56	12	15.75
*Thalamus*	L		−20	−24	16	15.32
*Mid. frontal gyrus*	R	9	40	18	22	15.20
*Putamen*	L		−26	4	16	12.91
*Insula*	L	13	−46	2	14	11.05
*Sup. Frontal Gyrus*	L	6	2	−6	50	10.15
*Insula*	L	13	−36	−18	18	10.06
*Cuneus*	R	17	−24	−82	12	9.20

Pos., posterior; Mid., middle; Sup. superior; L, left; R, right; BA, Brodmanns area. *^a^* FDR, 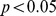 (random-effect analysis).

when different state of consciousness where compared in pairs only wake versus deep sedation showed significant differences (F-contrast, FDR, 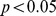). The same contrast was performed using an small volume correction (16 mm, p<0.001) centered in the group peak coordinates. In this case, only evidence of deactivation in STG (z-score  = 3.5) was found. Finally, sensory activation was investigated in the recovery state using an small volume correction (16 mm, p<0.001) centered in the HG coordinates. For this case, evidences of activations were found (z-score  = 4.17).

### Model level inference

**Figure 3:**
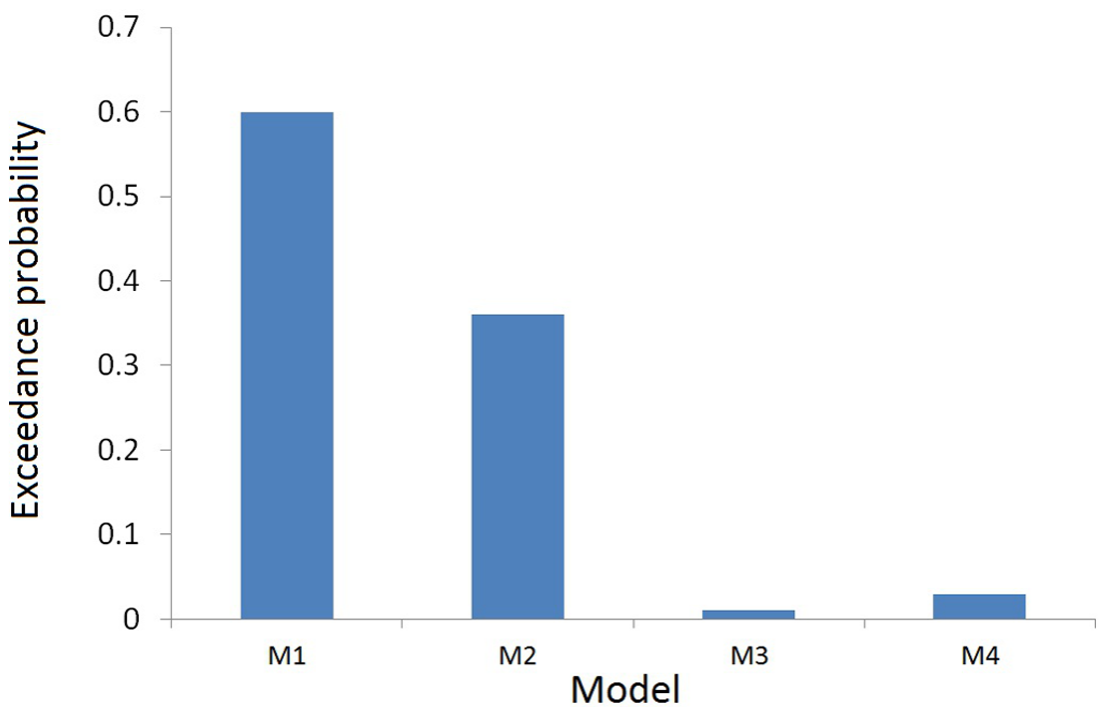
Random Effect Bayesian Model Selection (RFX-BMS) for selecting the best architecture across the different sedation levels. Two different models were selected indicating that there is no a unique model that explain the four states of consciousness.

First, we addressed the problem of determining the most plausible DCM family for wake and propofol-induced sedation states. In the four states of consciousness stochastic one-state models outperformed other DCM implementations (see Text S1 and Table S1). Then we turned to a model level inference regarding effective connectivity architecture. The results of the RFX-BMS for the winner family (ST1) for selecting the best architecture across the sedation levels are reported in figure 3.

**Figure 4:**
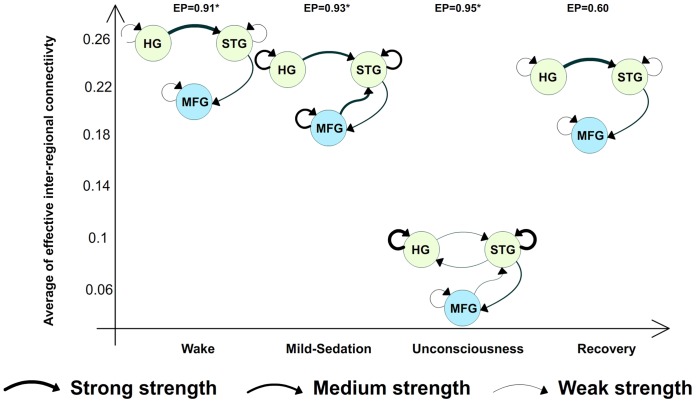
Model level inference in the different consciousness states using the stochastic one-state (ST1) models. The winner model for each state of consciousness is showed (EP, exceedance probability). Three different arrow sizes were used to illustrate different strength relevancies: strong (s), medium (m) and weak (w). For each connectivity parameter we used different thresholds on the average of the connectivity strength to assign a amplitude level: 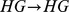 (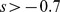, 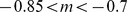 and 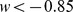) 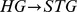 (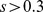, 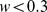), 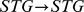 (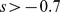, 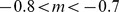 and 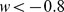), 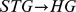 (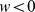), 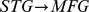 (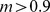 and 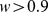), 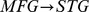 (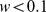). To illustrate the changes in effective connectivity the models were plotted versus the average of the absolute value of the strengths of inter-regional connections for the winner model in each state of consciousness.

As observed, two models can be selected M1 (p = 0.60) and M2 (p = 0.36) with high probability. This indicates that among the studied hypotheses there was not a unique model that explains the four states of consciousness. Then an additional RFX-BMS was performed to test the possibility of the use of different models in the different states of concioussness. The numerical results of this BMS for the winner family (ST1 family) are summarized in figure 4.

During the wake state a significant evidence in favor of model M1 was observed (exceedance probability, p = 0.91), as compared to any other model. For mild-sedation, the most likely model was M2 (p = 0.93). During unconsciousness, the optimal model was M4 (p = 0.95). This model corresponds to a completely connected architecture. Finally, M1 was found to be the winner model again during the recovery state (p = 0.60). Figure 4 also shows the drop in the average of the absolute value of the connectivity strength (only inter-regional connections) for the selected models during unconsciousness. This average of connectivity was computed on the winner model for the complete set of subjects. The model M4 during anesthesia was characterized by a significant decrease in the average connectivity strength 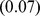 compared with the model M1 in wake state 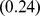 (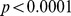).

To illustrate the relevancy of family model choice we also performed BMS on the classic DCM family (DT1) and we obtained that 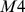 was the winner model for all consciousness states (wake p = 0.63, mild-sedation p = 0.70, unconsciousness p = 0.47 and recovery p = 0.55).

### Parameter level inference

**Figure 5:**
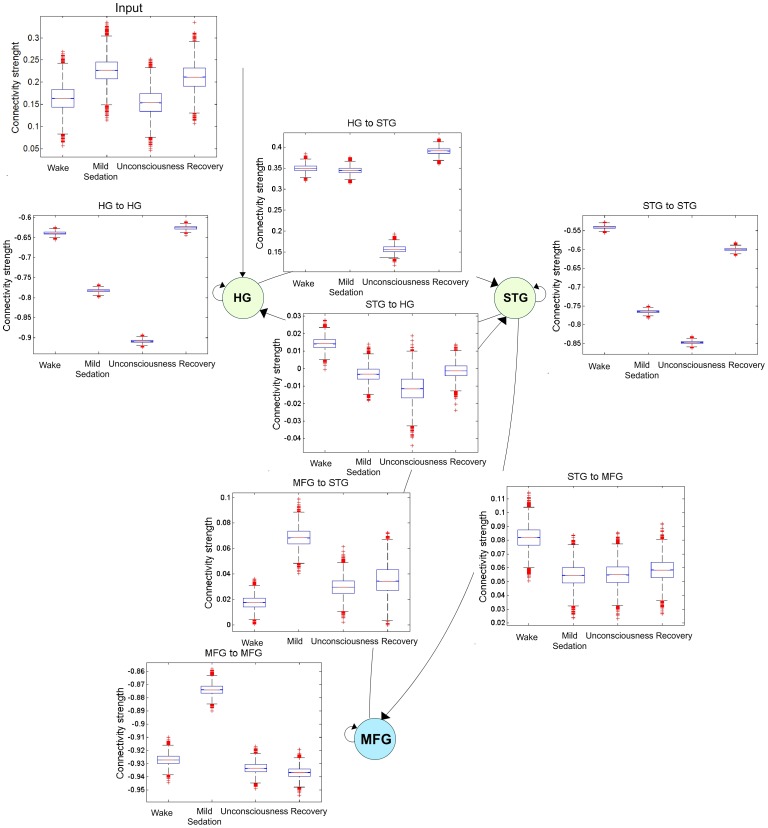
Parameter estimates under propofol. For each connection the box-and-whisker plot for 10000 samples drawn from each distribution of the strengths for the connectivity parameters in the four states of consciousness. The central mark corresponds the median, the edges of the box are the 25th and 75th percentiles and the whiskers are drawn from to illustrate the ends of the interquartile range.

Figure 5 shows the connectivity strengths estimated by the BMA procedure for each consciousness state. All connections were significant (posterior probabilities 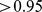) except for 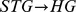 connection in mild-sedation 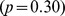 and recovery 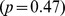. There was no significant difference in the connectivity strength for the input stimuli across the states of consciousness. For the anesthesia effect, there was an increase-recovery effect in self-inhibitory connections in regions located in the sensory cortex (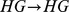 and 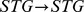, contrast 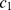, 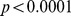). Noteworthy, the self-inhibitory activity was related to large negative values.

A similar effect, that is a decrease and then recovery of connectivity, was observed for connectivity parameters connecting regions located in auditory areas (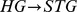 and 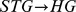, 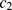, 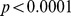). Connections related with the high functional order area MFG showed a significant increase in the connectivity strength during mild-sedation, both for the self-inhibitory (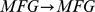, 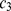, 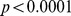) and the backward connection (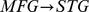, 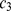, 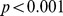). In summary, propofol loss of consciousness effect was linked to an increase of the self-inhibitory connectivity and a decrease of effective connectivity in the regions located in somatosensory cortices.

## Discussion

We presented here a DCM study of the effect of the anesthetic agent propofol on effective connectivity during mild sedation and unconsciousness. From the quantitative point of view, we observed significant increases in all self-inhibitory states during unconsciousness compared to wakefulness. This finding is consistent with local inhibitory changes predicted by other propofol models and in-vivo and in-vitro measurements [45], [53], [54]. We observed also decreases in the effective connectivity strengths for forward and backward connections in regions located in the auditory cortex. This finding is in line with predictions performed by several consciousness theories (thalamo-cortical breakdown [55], cortical cognitive unbinding [56], global neuronal connectivity breakdown and reduced integration information [57], [58]). This result is also consistent with previously reported breakdowns in functional connectivity [11], [14], [15]. In comparison with previous studies, our analysis strategy disentangles local (self-connections) and large range connectivity effects (connections between different cortical regions). We hypothesize that these changes can be associated to the high inhibitory activity resulting from the promotion of 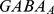 receptor activity by propofol [59], but possibly also to an effect on NMDA-mediated neurotransmission, known to be involved in the reciprocal influence of distant brain regions on their actual activity [9].

In this study, we focused our efforts on building accurate prior models to capture possible propofol-derived physiological changes. We constructed our models using four families (deterministic & one-state (DT1), deterministic & two-state (DT2), stochastic & one-state (ST1) and stochastic & two-state (ST2)), which we believe can capture the particular dynamics observed during propofol sedation [23], [47]. Our results indicate that, in this case, the stochastic family outperforms the deterministic one. A possible explanation is that stochastic models provide a formal treatment of internal fluctuations of the system, such as stochastic properties of sensory inputs or influence from subcortical (thalamic) regions not otherwise explicitly modeled [60]. These fluctuations can be of paramount importance, particularly during altered conscious states [60]. Our BMS results also suggest that stochastic modeling can improve the sensitivity for detecting different architectures of processing [61]. The BMS in the DT1 family provided very low exceedance probabilities for all the models we tested, i.e., there was no argument to prefer any specific model. In contrast, using the ST1 family provided high probabilities of model selection, even during propofol-induced alteration of consciousness. Interestingly, during the recovery state a low exceedance probability was observed, this could be explained by the greater inter-subject variability expected during this phase [62].

The decrease observed in self-connectivity in HG and STG during sedation suggests some kind of GABA-based hypothesis of the effects of propofol [63], [64]. However, this effect was not observed in MFG. Several hypotheses can be formulated to explain this observation. First, changes in self-connectivity in MFG during unconsciousness are still present but are relative to mild-sedation. MFG is a higher cognitive region and has the tendency to process the auditory stimuli. Hence at the level of mild sedation, while propofol try to decrease the neuronal activity, this area have the tendency to process the auditory stimuli resulting in activation of the MFG. Net result at the mild sedation level are the increases in self-connectivity in MFG and the feedback strength from MFG to STG (Figure 5). At the level of unconsciousness this tendency to process the auditory stimuli is no longer present (sedative unconsciousness – Ramsay 5) and hence self-connectivity activity on the MFG decreases as a result feedback input to STG also decreases. Therefore, when compared changes in self-connectivity they are still there but are relative to mild-sedation. A second explanation would be differences in sensibility to the propofol effect among primary, secondary and higher order area. In general, primary and secondary areas seem to be less sensitive to the propofol effect [7], [8]. For these cases large drops in self-connectivity maybe expected to affect these areas. In contrast, higher order areas exhibit high sensibility to the propofol effect [7], [65]. Therefore, maybe slightly weak self-connectivity changes may be enough to affect these regions.

During an alteration of consciousness, a “disconnection” between nodes will be expected [57], [58]. We found evidence of a reducing coupling effect as decreases of effective connectivity strengths (Figure 5). Importantly, in DCM there is no any specific modeling of these disconnections [17]. Our results are limited to discrete steady states of consciousness reached by keeping fixed the anesthetic dose during the record after the Ramsay evaluation. In the future, the connectivity changes herein observed will be further investigated at the subject level, for instance, by studying the full consciousness process in a single record using the propofol dose as a modulator parameter of connections. In this case, probably the architecture is not fundamentally changed, it is rather the intensity of the connection the one that is modulated (increased/decreased) by the propofol dose [66]. Testing of this new hypothesis could require a complex modeling because additional auditory and somatosensory stimuli coming from the Ramsay evaluation need also to be considered.

DCM for EEG has been recently used to study loss of consciousness in disorder of consciousness patients [49]. This study showed that by assuming a single model at the full population level (i.e., mixing both consciousness and unconsciousness subjects), it is possible to obtain reliable correlates of consciousness, in this case related to decreases in connectivity strength for top-down connections. In our model selection we tested this assumption of a common dynamic across the different states of consciousness. However, there was not a single winner model for the full population (figure 3), suggesting that probably for this case different states of consciousness can be explained by different dynamics and not by a single common model. This observation was confirmed by selecting the model independently in each state of consciousness. In this case, BMS furnished strong evidence that different models explain different states of consciousness (figure 4). Functional connectivity decreases have been also linked to loss of consciousness during propofol sedation [14], [15]. These studies suggest loss of consciousness is linked to a decrease in the capacity of the brain to integrate information, in thalamo-cortical, low to high cortices connections and within fronto-parietal areas. This information keeps descriptive, because there is no description of mechanisms responsible of these breakdowns. In this study, we used a model based approach to investigate the breakdown between low auditory cortices and high level cortices in response to an auditory external stimulus. Our results suggest the breakdown mechanism is not only related to the communication path itself, but also with the self-inhibitory process. Similar investigations can be performed to study the breakdown process for other sensory modalities. Nevertheless, extension of these findings to the other communication breakdowns typically observed during unconsciousness is not straightforward. In particular, the DCM for thalamo-cortical connection will require a better understanding of the neuronal dynamic and the hemodynamic response function for the thalamus [67]. In other hand, modeling of breakdowns in fronto-parietal areas is a challenge from the DCM perspective, because the difficulty to perturb the fronto-parietal during unconsciousness states [68]. An alternative that can cover simultaneously all these mechanisms can be the network discovery using endogenous activity in task-free (resting state), however, these methods are still under investigation [69].

Propofol induced unconsciousness provides a pharmacological model to study the complex phenomena of loss-of-consciousness in experimentally controlled way. In this study we investigated the changes in effective connectivity in different states of consciousness using propofol as a prototype model to induce the loss of consciousness. Mechanisms of induction of loss-of-consciousness are highly diverse and heterogeneous and in most of the cases this phenomena cannot be explicitly controlled, as in the pharmacological scenario herein studied [1], [31]. The main aim of our study was to build hypotheses about this loss-of-consciousness phenomenon in terms of the effective connectivity. But also to provide general explanations of the unconsciousness phenomena that can be translated to other less controlled loss-of-consciousness scenarios, such as, disorders-of-consciousness or epileptic seizures. In this work the experimental variable that induces the loss-of-consciousness was not explicitly considered as part of the causes of the observed dynamic. This modeling selection results in a “first order’’ family of models that provides explanation to the loss-of-consciousness process without the need of any explicit modeling of the underlying cause of the unconsciousness. Remarkably, under this very general assumption the proposed modeling approach was able to highlight significant differences at architectural level between different states of consciousness. Alternatively, a more complex “second order” model including explicitly the perturbation mechanism can also be used to explain the brain dynamics during unconsciousness states, including resting blocks. For instance, the level of propofol plasma concentration can be used as modulation parameter in the DCM. This specialized model probably cans also highly changes in effective connectivity. Nevertheless, it is worthy to observe that these higher order models can be hardly to translate to less controlled loss-of-consciousness scenarios. Since it require the knowledge of the experimental input than induce unconsciousness, an uncontrolled parameter in less other loss-of-consciousness scenarios. In summary, the proposed model provides a first order approximation to the loss-of-consciousness dynamic resulting in a general model with the drawback of not including explicitly the pharmacological effect of propofol. In contrast, a second order model would model explicitly this input. However, this last modeling strategy will lack of generalization capacity to explain the unconsciousness phenomena in less controlled scenarios. In terms, of our initial goal of generalization we considered that first order models provide a good compromise between complexity and fitting.

Stochastic DCM provides a strategy to isolate noise coming from random fluctuations from the deterministic DCM structure. These random fluctuations correspond, for instance, to endogenous fluctuations that arise from self-organised dynamics [16], [69]. Recent works in real and simulated data comparing stochastic and deterministic DCM support the relevancy of these fluctuations for the neuronal dynamic generation [70]. A potential risk of this approach can be the under-estimation of the effective connectivity parameter. This is because in principle, the effective connectivity information can be completely explained as endogenous random fluctuations. In our experiments the parameters that control the level of noise keep fixed around the initial prior and across different states of consciousness (data not shown). This suggests that noise is not over explaining the data and the remaining information, not adjusted by the noise, would be captured by the deterministic part. In addition, even in the unconsciousness state which is characterized by a low signal-to-noise-ratio, effective connectivity parameters were still significant, i.e., the model keeps the capacity of capturing neuronal interactions in his deterministic part. In summary, by controlling the level of noise the risk of the effective connectivity under-estimation is reduced and the conclusions derived from our data will be still valid.

The hypotheses herein addressed are related to a simple and well-established dynamic derived from an auditory processing task. This dynamic was studied under different experimental conditions related to the level of consciousness. By using this approach the observed changes in effective connectivity can be directly related to this well-established dynamic. A limitation of this approach is that the observed changes are conditioned to the studied dynamic. Therefore, additional effects of the propofol maybe not modeled. An alternative approach to overcome this issue would be the direct study of the propofol effect by modeling a new dynamic including regions directly affected by the anesthetic. For instance, one can consider models for the propofol effect that include some of the regions that showed the main effect of propofol sedation (wake > sedation, see table 3). In principle, these models account for the main effect of the region suppression. However, it is worthy to point out that this DCM study requires a-priori definition of the architecture of connectivity. This maybe a difficult requirement to achieve in propofol, where there is no enough prior knowledge to establish these new hypotheses. In any case, more sophisticated models accounting explicitly for the propofol effect must be further investigated.

The hypotheses herein addressed are related to a simple and well-established dynamic derived from an auditory processing task. This dynamic was studied under different experimental conditions related to the level of consciousness. By using this approach the observed changes in effective connectivity can be directly related to this dynamic. A limitation of this approach is that the observed changes are conditioned to the studied dynamic. Therefore, additional effects of the propofol maybe not modeled explicitly. An alternative approach to overcome this issue would be the direct study of the propofol effect by modeling a new dynamic including regions directly affected by the anesthetic. For instance, one can consider models for the propofol effect that include some of the regions that showed the main effect of propofol sedation (wake > sedation, see table 3). In principle, these models account for the main effect of the region suppression. However, it is worthy to point out that in this case the DCM study would requires a-priori definition of the architecture of connectivity. A difficult requirement to achieve in propofol, where there is no enough prior knowledge to establish these new hypotheses. In the future, more sophisticated models accounting explicitly these effects must be further investigated.

Finally, the accuracy of the DCM-generated models deserves some discussion [47], [61]. The two mechanisms herein modeled (stochastic and two-sate) have been proven to be relevant for the understanding of the hypnotic effect of anesthesia [45], [71]. Our results indicate that stochastic fluctuations are more relevant to the modeling of neuronal dynamics during altered consciousness states than inhibitory-excitatory effect modeling. The inhibitory-excitatory approach herein used aims at explicitly modeling glutamatergic (excitatory) and GABAergic (inhibitory) subpopulations simply, by connecting two additional states and changing their connectivity priors. Even if this explicit model can improve the data fitting, this improvement does not compensate for the increase in complexity introduced by the two additional states used for each neuronal region. In the future, more sophisticated models should be specifically designed to study anesthesia-induced alteration of consciousness. They should take account anesthetic agent concentration [66]. Similarly, more specialized models could also be considered for the modeling of (un)consciousness as a whole. For example, those models should take into account the thalamic connections, which were not considered in this work because of the absence of strong activation in the thalamus, and interactions with autonomous oscillatory systems involved in unconsciousness generation, such as modulations within the default mode network [72].

In summary, in the present study we showed that the communication breakdown observed in low to high cortices connections in response to external auditory stimuli during propofol-induced unconsciousness is the result of a mixture of two effects: a local inhibitory connectivity increase and a decrease in the effective connectivity in sensory cortices. Our findings extend previous studies that show communication breakdown during resting state by providing a mechanistic explanation of how the disconnection is emerging. In addition, we also verified the relevance of the stochastic fluctuations as a key feature for the modeling of (un)consciousness. Further studies are needed to confirm these results in other sensory stimulus and other communication breakdowns observed in unconsciousness.

## Supporting Information

Text S1 **Family level inference.**
(PDF)

Table S1 **Estimated exceedance probabilities for different fMRI-DCM families in each consciousness state.**
(PDF)
