# Supplementary material for: Changes in Effective Connectivity by Propofol Sedation
Source: PLoS One. 2013 Aug 19;8(8):e71370. doi: 10.1371/journal.pone.0071370 (PMC3747149; doi:10.1371/journal.pone.0071370)
Supplement: Text S1 — Family level inference. (PDF) [file pone.0071370.s001.pdf]

S1 Text

S1.1 Family level inference

We addressed the problem of determining the most plausible DCM family for wake and propofol-induced sedation states. See Table S1 for the results.

This probability corresponds to the assumption that a family k is more likely than any other (of the K families compared), given the data from all subjects [1]. As observed, the family of stochastic models with one-state per region was more likely than the other compared families in all consciousness states (exceedance probability, $p > 0.99$). With high confidence ($p > 0.99$) the stochastic models outperformed deterministic models in the complexity/fitting measurement (free-energy). As observed, the most complex models with two-states per region did not provide the best explanation for the experimental data.

References

[1] Penny W, Stephan K, Daunizeau J, Rosa M, Friston K, et al. (2010) Comparing families of dynamic causal models. PLoS Comput Biol 6: e1000709.
