# Supplementary material for: Changes in Effective Connectivity by Propofol Sedation
Source: PLoS One. 2013 Aug 19;8(8):e71370. doi: 10.1371/journal.pone.0071370 (PMC3747149; doi:10.1371/journal.pone.0071370)
Supplement: Table S1 — Estimated exceedance probabilities for different fMRI-DCM families in each consciousness state. (PDF) [file pone.0071370.s002.pdf]

S1 Table

	Wake	*Mild-sedation*	*Unconciousness*	*Recovery*
Deterministic - One state	0.0003	0.0005	0.0005	0.0006
Deterministic - Two states	0.0003	0.0001	0.0004	0.0004
Stochastic - One state	0.9986	0.9989	0.9987	0.9984
Stochastic - Two states	0.0008	0.0005	0.0004	0.0006

Table S1: Estimated exceedance probabilities for different fMRI-DCM families in each consciousness state.
